# Are rates of pediatric bipolar disorder increasing? Results from a nationwide register study

**DOI:** 10.1186/s40345-014-0010-0

**Published:** 2014-09-16

**Authors:** Lars Vedel Kessing, Eleni Vradi, Per Kragh Andersen

**Affiliations:** Psychiatric Center Copenhagen, Department O, 6233 and University of Copenhagen, Blegdamsvej 9, Copenhagen, 2100 Denmark; Department of Biostatistics, University of Copenhagen, Copenhagen, 1165 Denmark

**Keywords:** Mania, Bipolar disorder, Child and adolescents, Youth, Incidence rates

## Abstract

Studies from the USA suggest that rates of pediatric bipolar disorder have increased since the mid-90s, but no study outside the USA has been published on the rates of pediatric bipolar disorder. Further, it is unclear whether an increase in rates reflects a true increase in the illness or more diagnostic attention. Using nationwide registers of all inpatients and outpatients contacts to all psychiatric hospitals in Denmark, we investigated (1) gender-specific rates of incident pediatric mania/bipolar disorder during a period from 1995 to 2012, (2) whether age and other characteristics for pediatric mania/bipolar disorder changed during the calendar period (1995 to 2003 versus 2004 to 2012), and (3) whether the diagnosis is more often made at first psychiatric contact in recent time compared to earlier according to gender. Totally, 346 patients got a main diagnosis of a manic episode (F30) or bipolar affective disorder (F31) at least once during the study period from 1995 to 2012. For both sexes, annual rates of mania/bipolar disorder two to four doubled during the study period (0.001% before year 2004 to 0.002%–0.004% in 2010). Median age at the index diagnosis was very similar during the two calendar periods (17.2, quartiles, 16.2–18.3 versus 17.4, quartiles, 16.1–18.2) indicating that the diagnosis of mania/bipolar disorder was not made earlier in the recent calendar period. Similarly, there were no differences between early versus late in the study period in the fractions of first contact diagnosis of mania/bipolar disorder diagnoses, the contact number at which patients got the diagnosis or the duration from first psychiatric contact to the diagnosis of mania/bipolar disorder. The rate of diagnosis of mania/bipolar disorder increased from 1995 to 2014, which did not seem to be explained by more diagnostic attention.

## Background

An influential paper by Wozniak and colleagues raised in 1995 the question of whether pediatric bipolar disorder was being overlooked in clinical practice (Wozniak et al. 1995). Since then, clinical attention toward pediatric bipolar disorder has increased substantially, and concerns regarding missed diagnoses and underestimation of the prevalence have been replaced by concerns of misdiagnoses of bipolar disorder and overestimates of the prevalence (Goldstein 2012). Thus, studies from the USA have found increasing rates of inpatient psychiatric hospitalization (Blader and Carlson 2007; Lasky et al. 2011) and outpatient treatment rates (Moreno et al. 2007) during the last decade. Although it is a clinical experience that a similar tendency has taken place in Europe and elsewhere since 1995, no study outside the USA has been published on the rates of clinical pediatric bipolar disorder. Further, it is unclear whether a possible increase in the prevalence of the diagnosis of pediatric bipolar disorder reflects a true increase in the incidence of bipolar disorder or is a result of increased diagnostic attention. In a US cohort of patients with bipolar disorder, 61% reported onset during childhood or adolescence compared to 30% in cohorts from the Netherlands or Germany (Post et al. 2008). On the other hand, studies from the USA (Lish et al. 1994; Egeland et al. 2000; Post et al. 2010) and a study from Spain (Soutullo et al. 2009) suggest a delay in diagnosis of pediatric bipolar disorder so the diagnosis of bipolar disorder is only made following several contacts to medical psychiatric care. Thus, no study has investigated whether the increasing prevalence of pediatric bipolar disorder is explained by earlier age at which the diagnosis is made or whether the diagnosis is more often made at first psychiatric contact in recent time. If clinicians have been more observant on bipolar disorder in recent years, age at diagnosis should have decreased in recent times and the diagnosis of bipolar disorder should be made at earlier or even first contacts.

Using nationwide registers of all inpatient and outpatient contacts to all psychiatric hospitals in Denmark, the aim of the present study was to investigate (1) gender-specific rates of incident pediatric mania/bipolar disorder during a period from 1995 to 2012, (2) whether age and other characteristics for pediatric mania/bipolar disorder have changed during this calendar period, and (3) whether the diagnosis is more often made at first psychiatric contact in recent time compared to earlier according to gender.

## Methods

### The register

The Danish Psychiatric Central Research Register (DPCRR) is nationwide with registration of all psychiatric hospitalizations in Denmark for the 5.3 million inhabitants (Munk-Jorgensen and Mortensen 1997). From 1970 to 1993, ICD-8 was used, and up to 1995, only inpatient stay at psychiatric hospitals and wards was included. From January 1, 1995, the register included information on patients in psychiatric ambulatories and community psychiatry centers, also. Since January 1, 1994, the ICD-10 has been in used in the register (World Health Organisation 1993). General practitioners and private practicing psychiatrist do not report to the DPCRR. General practitioners are not allowed to treat children and adolescents with mania or bipolar disorder in Denmark in contrast to private practicing psychiatrists.

All inhabitants in Denmark have a unique person identification number (Civil Person Registration number, CPR-number) that can be logically checked for errors, so it can be established with great certainty if a patient has had contact to psychiatric service previously, irrespective of changes in name, etc.

No private psychiatric inpatient hospitals or department are in operation in Denmark; all are organized within public services and reporting to the DPCRR.

The Medicinal Product Statistics contains data on all prescribed medication purchased at pharmacies from January 1, 1995 and onwards (Danish National Board of Health 2002). In Denmark, all medications prescribed by doctors, such as lithium and anticonvulsants, are purchased only at pharmacies and the resulting data are electronically recorded in the Medicinal Product Statistics.

According to Danish law ethical approval is not required in register based studies.

### The sample

The study sample was defined as all children and adolescents <19 years with a contact as outpatient (patients in psychiatric ambulatories and community psychiatry centers) or inpatient (patients admitted during daytime or overnight to a psychiatric hospital) with at least one main diagnosis of mania/bipolar disorder (ICD-10, code DF30-31.9) during the study period from January 1, 1995 to December 31, 2012. Patients with psychiatric contacts prior to 1995 and back to 1970 were excluded.

### Statistical analysis

*Aim 1*. Annual rates of incident pediatric mania/bipolar disorder in children and adolescents were calculated from 1995 to 2012 separately for boys and girls using the entire Danish population of less than 19-year-olds as denominator.

*Aim 2*. The study period was divided into two 9-year periods: 1995 to 2003 and 2004–2012. Patients belonged either to the first or to the second group depending on the date of their first diagnosis of mania/bipolar disorder (the index diagnosis). Characteristics of patients during the two periods were compared.

In Table 1, the variable incidence rate of the first drug was calculated as the number of patients who got a given first drug divided by the person years at risk, separately for each calendar period for the drug. The variable rate of drug treatment was calculated as the number of prescriptions of a given drug from index bipolar to the end of the calendar period/19th birthday divided by person years.

**Table 1:** **Characteristics of children and adolescents with a first diagnosis of mania or bipolar affective disorder (index diagnosis)**

	**1995–2003**	**2004–2012**	***P*** **value**
*N* = 346 (%)	111 (32.1)	235 (67.9)	
Sex (%)			
Boys	46.8	41.7	0.4
Girls	53.2	58.3	
Age at index diagnosis, median (quartiles)	17.2 (16.2–18.3)	17.4 (16.1–18.2)	0.8
Contact number at index diagnosis, median (quartiles)	2 (1–4)	2 (1–4)	0.9
Duration from first psychiatric contact to first diagnosis of mania/bipolar disorder (years)	0.09	0.29	0.1
	(0–1.03)	(0–1.35)	
Bipolar index diagnosis (%)			
Single manic/mixed episode	40.5	25.5	0.005
Bipolar disorder	59.5	74.5	
Bipolar index diagnosis (%)			
Remission, other, unspecified	10.8	23.9	
Depressive	7.2	14.1	
Manic (including single manic episode)	70.3	48.3	0.0009
Mixed (including single mixed episode)	11.7	13.7	
Auxiliary diagnosis (%)			
No auxiliary diagnosis	84.7	66.0	0.0003
Psychiatric	11.7	21.7	0.03
Substance abuse	0.9	3.0	0.23
Somatic	8.1	17.0	0.03
Type of auxiliary psychiatric diagnoses (%)			
F4	15.4	27.5	
F5	0	2.0	0.77
F6	15.4	11.8	
F7–9	69.2	58.8	
Rate of drug treatment per person year after index diagnosis and before <19 years of age			
Lithium	3.6	1.1	<0.0001
Antipsychotics	3.6	5.3	<0.0001
Antiepileptics	1.7	2.7	<0.0001
Antidepressants	1.2	1.5	0.0125
Incidence rate per person year of first drug after index diagnosis and before <19 years of age			
Lithium	0.4	0.1	<0.0001
Antipsychotics	0.6	1.6	<0.0001
Antiepileptics	0.3	0.4	<0.0001
Antidepressants	0.2	0.3	0.389

According to calendar periods 1995–2003 versus 2004–2012.

*Aim 3*. For each calendar year, the fraction of first contact diagnosis of mania/bipolar disorder diagnoses out of all contacts diagnoses of mania/bipolar disorder was calculated for boys and girls.

Categorical data were analyzed with chi-square test, and continuous data were analyzed with the Mann-Whitney test for two independent groups. *P* < 0.05 was used to indicate statistical significance.

## Results

**Figure 1:**
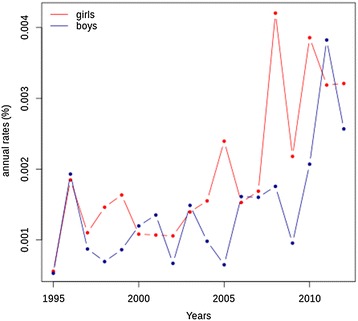
**Annual rates from 1995 to 2012 of incident mania or bipolar disorder for boys and girls.**

Totally, 346 patients got a main diagnosis of a manic episode (F30) or bipolar affective disorder (F31) at least once during the study period from 1995 to 2012. Figure 1 presents gender-specific annual rates of incident mania or bipolar disorder from 1995 to 2010. As can be seen, for both sexes, annual rates were rather stable around 0.001% before year 2004 and then increased to 0.002%–0.004% in 2010. This two to four doubling in annual rates from the first to the second calendar period was only to a very minor degree explained by an increase in the general population aged 13 to 18 years (i.e., the denominator). Thus, the fraction aged 13–18 in the general population among all children aged 0–18 years increased only from 28% in the first calendar period to 31% in the second calendar period.

Among the total 346 patients, 111 patients (32.1%) got the main diagnosis during the calendar period 1995–2003 whereas the 235 remaining patients (67.9%) got the diagnosis in the years 2004 to 2012. Table 1 presents characteristics of patients in the two calendar periods. As can be seen, there were no statistical significant differences in gender composition or age at index diagnosis (first diagnosis of mania or bipolar affective disorder). Median age at the index diagnosis was in fact very similar during the two calendar periods (17.2, quartiles, 16.2–18.3 versus 17.4, quartiles, 16.1–18.2) indicating that the diagnosis of mania/bipolar disorder was not made earlier in the recent calendar period. Similarly, there were no differences in the contact number at which patients got the index diagnosis or in duration from first psychiatric contact to the index diagnosis.

As can be further seen from Table 1, significantly and substantially more patients got a diagnosis of bipolar disorder during the calendar period from 2004–2012 (74.5% versus 59.5%). Thus, these patients had had at least one other affective episode prior to the index diagnosis of bipolar disorder, and this or these episodes were either untreated or treated in primary care by a private psychiatrist (as general practitioners do not treat these patients). This is further substantiated as during 2004–2012, more patients were in a remitted, unspecified, or depressive state when the index diagnosis was made, indicating that they had suffered from a hypomanic or manic episode prior to their first hospital contact.

More psychiatric comorbid diagnoses were made during 2004–2012 than during 1995–2003, 21.7% versus 11.7%, but the type of psychiatric comorbid diagnoses did not differ significantly between the two calendar periods.

During the recent calendar period, lithium was prescribed more rarely after the index diagnosis in general and as first drug and conversely, antipsychotics and anticonvulsants were used more often. For example in the calendar period 1995–2003, the rate of lithium treatment after the index diagnosis was 3.6 prescriptions per person year, which was calculated as the number of lithium prescriptions from the index diagnosis to the end of the calendar period/19th birthday (*N* = 635) divided by person years (177.28 person years). Similar, the incidence rate of lithium as first drug was 0.4 per person year corresponding to 40 patients who got lithium per 100 person years, which was calculated as the number of patients who got lithium (*N* = 46) divided by the person years (105.81) for the drug.

**Figure 2:**
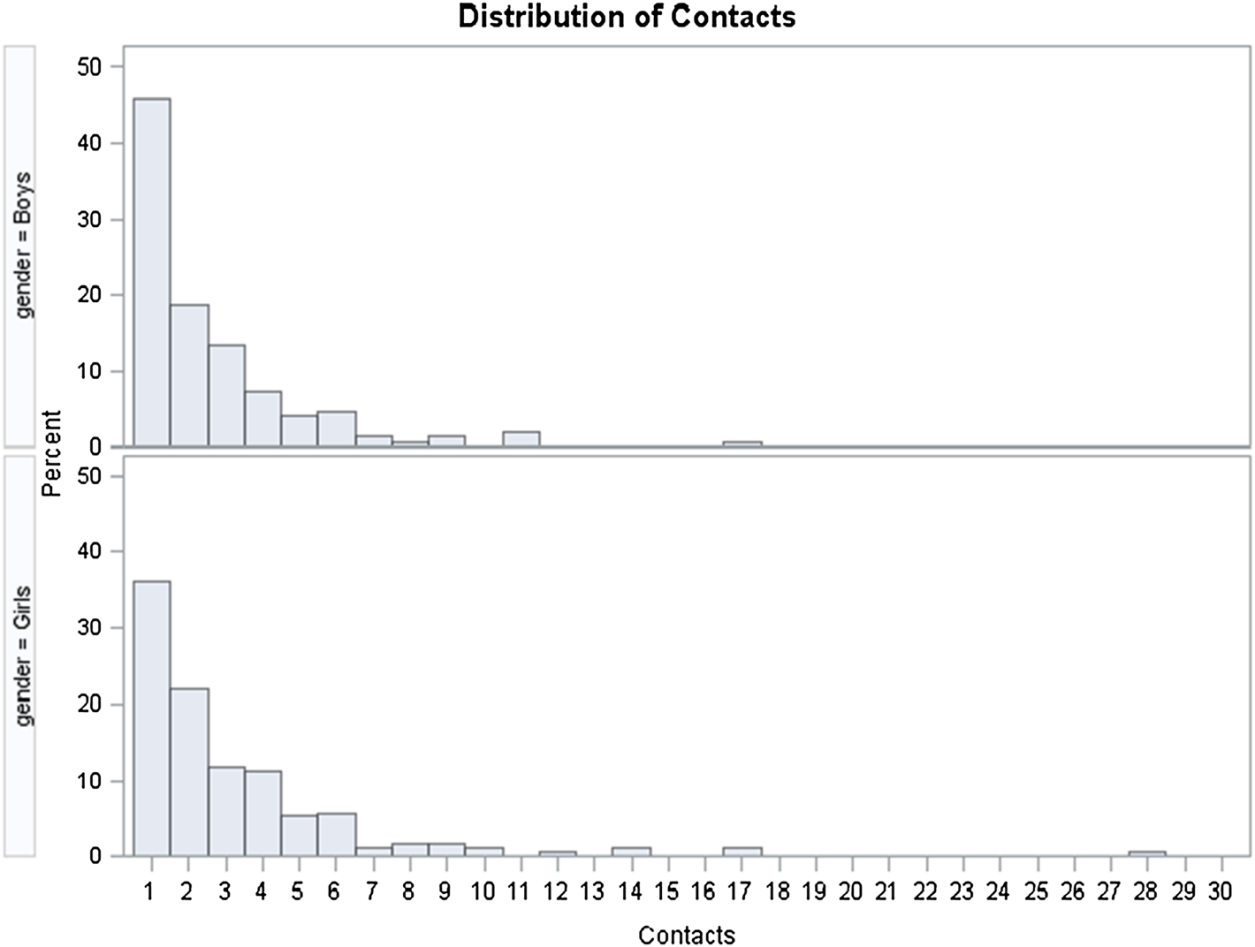
**The contact number at which patients got the diagnosis of mania/bipolar disorder for the first time.**

**Figure 3:**
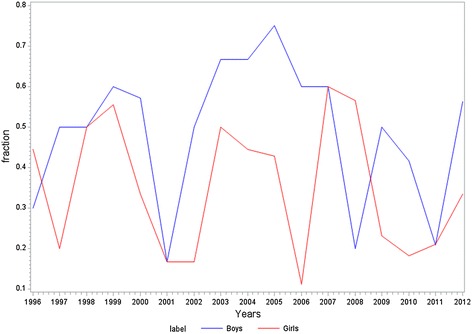
**Fraction of first contact diagnosis of mania/bipolar disorder diagnoses for boys and girls.**

Figure 2 illustrates that the contact number at which patients got the diagnosis of mania/bipolar disorder for the first time did not differ for boys and girls, and Figure 3 shows that the fraction of first contact diagnosis of mania/bipolar disorder diagnoses did not increase from 1996 to 2012 for boys and girls, indicating that within hospital-based psychiatry, the diagnosis of mania/bipolar disorder was not made at earlier contacts in more recent times.

## Discussion

This is the first study outside the USA that has investigated population-based annual rates of incident pediatric bipolar disorder during the last decades and, further, the first study aiming to test whether a possible increase in the incidence is a result of increased diagnostic attention during recent times. For both sexes, annual rates of mania/bipolar disorder two to four doubled during the study period from 1995 to 2012, and this increase did not seem to be explained by increased diagnostic attention. Median age at the index diagnosis was very similar during the first half calendar period - 1995 to 2003 and the second half calendar period - 2004 to 2012 (17.2, quartiles, 16.2–18.3 versus 17.4, quartiles, 16.1–18.2) indicating that the diagnosis of mania/bipolar disorder was not made earlier in the recent calendar period. Similarly, there were no differences between early versus late in the study period in the fractions of first contact diagnosis of mania/bipolar disorder diagnoses, the contact number at which patients got the diagnosis or the duration from first psychiatric contact to the diagnosis of mania/bipolar disorder. Indeed, it is unlikely that increased diagnostic attention in primary care would not have led to a change in these parameters in psychiatric health care service.

The study included a Danish nationwide sample of all in- and outpatients below 19 years of age with an outpatient or inpatient psychiatric hospital contact with a diagnosis of mania/bipolar disorder in psychiatric settings. In Denmark, doctors are obliged to make a diagnosis when a treatment period is terminated, i.e. at discharge from hospital or at the end of an ambulatory treatment period, and all diagnoses are reported to the Danish Psychiatric Central Research Register.

Overall, our study including a Danish sample confirm the increase in rates of inpatient psychiatric hospitalization (Blader and Carlson 2007; Lasky et al. 2011) and outpatient treatment rates (Moreno et al. 2007) during the last decade in the USA and extend this phenomenon to a European sample. On the other hand, this finding contrasts the result of a meta-analysis of epidemiological studies of pediatric bipolar disorder showing no relation between the year of data collection and prevalence rate across studies (Van Meter et al. 2011). However, these results may be considered less valid as the analysis was based on cross-sectional data of prevalence (and not incidence) of a diagnosis of pediatric bipolar disorder and a comparison between studies using different diagnostic methods.

In our study, the finding of increased incidence of pediatric bipolar disorder diagnoses did not seem to be explained by increased attention to bipolar disorder among clinicians and an earlier age at first diagnosis as the high prevalence of onset during childhood or adolescents (30%–60%) found in recent studies might suggest (Post et al. 2008). There were no differences between early versus late in the study period in age at index diagnosis, the fractions of first contact diagnosis of mania/bipolar disorder diagnoses, the contact number at which patients got the diagnosis, or the duration from first psychiatric contact to the diagnosis of mania/bipolar disorder. Indeed, it is unlikely that increased diagnostic attention in primary care (general practitioners or private psychiatrists) or within psychiatric hospital service would not have led to a change in these parameters. On the other hand, during the study period, significant changes in diagnostic habits or treatment-seeking behavior of subjects took place. The proportion of patients who got an index diagnosis of a single manic or mixed episode decreased during the study period from 40.5% to 25.5%, and the proportion of bipolar disorders diagnosed in manic episodes decreased from 70.3 to 48.3% whereas the proportion diagnosed in remission or depressive episodes increased from 10.8% to 23.9% and 7.2% to 14.1%, respectively (Table 1). These changes in the bipolar index diagnosis could at least partly be an artifact as more patients in the first calendar period may have had a psychiatric outpatient hospital contact, which was not recorded in the Danish Psychiatric Central Research Register prior to 1995. Alternatively, one might suggest that these changes in diagnostic habits or treatment-seeking behavior could reflect a higher proportion of patients being diagnosed with bipolar disorder, type II, during the more recent calendar period. For example, it is possible that patients more frequently might have sought help for depressive or mixed episodes or comorbid disorders during the second calendar period, which finally led to the diagnosis of a bipolar disorder in depression or remission. However, again, it is unlikely that such increased attention to bipolar disorder could have occurred without changes in age at index diagnosis, the fractions of first contact diagnosis of mania/bipolar disorder diagnoses, the contact number at which patients got the diagnosis, or the duration from first psychiatric contact to the diagnosis of mania/bipolar disorder.

The prescription pattern of drugs also changed during the study period (lithium was prescribed more rarely and antipsychotics and anticonvulsants more often in the recent calendar period, Table 1) but it is not clear how this relates to diagnostic habits or treatment-seeking behavior of subjects.

In summary, our findings should be interpreted with caution. Based on the results, we cannot exclude that the increasing annual rates of incident clinical pediatric bipolar disorder reflects a true finding of increasing incidence of pediatric bipolar disorder—on the other hand, the study cannot confirm this either.

### Advantages of the present study

The study comprises an observation period of up to 16 years of the whole Danish population (5.3 million inhabitants out of whom approximately 1,175,201 (22.2%) are less than 19 years old), and further, the population is ethnically and socially homogeneous and with a very low migration rate. The entire population (approximately 100%) of patients treated in psychiatric settings in a whole country during in- or outpatient settings was included. Psychiatric care is well developed in Denmark, so persons with mania or bipolar disorder can easily come in contact with psychiatric community centers or hospitals. Also, as psychiatric treatment in Denmark is free of charge, the study is not biased by socioeconomic differences.

### Limitations of the present study

It should be noted that the study included patients who have passed the threshold for treatment to psychiatric outpatient settings (psychiatric ambulatories and community centers) or to psychiatric hospitalization, only. Although the vast majority of children and adolescents with bipolar disorders are treated in these hospital settings as in- or outpatients, some patients with milder types of the illness may be treated within private psychiatric practice. Such patients are not included in the study, as private psychiatric practice does not report to the DPCRR. General practitioners do not treat children and adolescents with bipolar disorder in Denmark. It cannot be excluded that including milder cases of bipolar disorder into the sample might have resulted in other findings.

It should be noted that the diagnosis of bipolar disorder in ICD-10 includes both bipolar disorder I and bipolar disorder II but does not discriminate between the two subtypes, as ICD-10 bipolar disorder is defined as a disorder with at least two mood episodes among which at least one is a hypomanic or a manic episode. It is most likely that the majority of the 346 patients in the present study suffered from bipolar disorder, type I, as patients were included via their contact to hospital psychiatric settings. It is most likely that patients with bipolar disorder, type II were not included as also reflected in the low incidence rates of 0.001% to 0.004% compared with 12-month prevalence rates for mania with and without depression of 2.2% and 1.3% from a recent population-based US survey of 13–18-year-old adolescents (Merikangas et al. 2012). In this way, our findings may be generalized to patients with bipolar disorder, type I, but not to bipolar disorder, type II.

It should further be noted that the median age at the index diagnosis was rather high (17.2 years) and that only 25% of the patients were below 16.2 years of age when the diagnosis of mania/bipolar disorder was made for the first time reflecting that child bipolar disorders are rarely diagnosed in Denmark.

Frequent comorbid conditions in bipolar disorder such as anxiety disorders, hyperkinetic disorders, and substance use may complicate the diagnostic process. It should be stressed that the present study focused on the main diagnoses that are given for the main illness leading to investigation and treatment. The main diagnoses are given according to the diagnostic hierarchy in ICD-10 giving priority to diagnoses with lower ICD-10 codes (World Health Organisation 1992). According to the diagnostic guidelines, a comorbid illness should be recorded as an auxiliary diagnosis only when the comorbid illness is independent of the primary illness. Auxiliary diagnoses are seldom recorded in Denmark.

## Conclusions

In Denmark, annual rates of mania/bipolar disorder two to four doubled for both sexes during a study period from 1995 to 2012, and this increase did not seem to be explained by increased diagnostic attention.
